# Optimizing Extraction, Evaluating Antioxidant Activity, and Analyzing Bioactive Compounds in Trikaysornmas Formula

**DOI:** 10.1155/2024/8335536

**Published:** 2024-09-18

**Authors:** Suphatson Limsakul, Orawan Monthakantirat, Yaowared Chulikhit, Juthamart Maneenet, Charinya Khamphukdee, Yutthana Chotritthirong, Achiraya Phasomsap, Chantana Boonyarat, Supawadee Daodee

**Affiliations:** ^1^ Division of Pharmaceutical Chemistry Faculty of Pharmaceutical Sciences Khon Kaen University, Khon Kaen 40002, Thailand; ^2^ Natural Drug Discovery Laboratory Institute of Natural Medicine University of Toyama, 2630 Sugitani, Toyama 930-0194, Japan; ^3^ Division of Pharmacognosy and Toxicology Faculty of Pharmaceutical Sciences Khon Kaen University, Khon Kaen 40002, Thailand

## Abstract

The Trikaysornmas formula (TKM) represents a prevalent Thai traditional remedy utilized extensively in Thailand. Its traditional uses include appetite enhancement, functions as a nourishing tonic, and exhibits adaptogenic properties. Comprising *Aegle marmelos* fruit, *Nelumbo nucifera* stamen, and *Jatropha multifida* bark, this formula embodies the synergy among these three herbs. The objective of this study was to optimize the extraction method, determine the active compounds in the TKM, and evaluate its antioxidant activity. The optimization of the extraction method for this formula was studied using an experimental design. Phytochemical components such as total phenolics, total flavonoids, total carotenoids, and total alkaloids were assessed utilizing a colorimetric method. Antioxidant activities were assessed through DPPH free radical scavenging, ABTS radical cation decolorization, oxygen radical absorbance capacity, ferric reducing antioxidant power, metal chelating activity, and lipid peroxidation assay. For the analysis of active constituents in the formula, gallic acid, kaempferol-3-o-glucoside, imperatorin, vitexin, and scopoletin, a validated reversed-phase column high-performance liquid chromatography (HPLC) method was developed. The total active contents including phenolic, flavonoid, carotenoid, and alkaloid compounds were found in the formula. The developed HPLC method exhibited reliable results in all validation parameters. TKM demonstrated antioxidant activity in the models used in this research. The findings from this study can serve as valuable tools for standardization and quality control measures. Additionally, they can contribute to maximizing the possibilities inherent in this traditional Thai formulation.

## 1. Introduction

Herbal medicine has been used for a long time, and it is a popular remedy used in healthcare system in Thailand. The consumption of plant-based medicine and other botanicals has increased in recent years. It involves using natural and biologically based practices and products to treat a variety of physical or emotional conditions [1]. Herbal products are widely used worldwide in many countries at present [2]. Thai traditional medicine has a rich history of incorporating local herbs that have been utilized for generations. This knowledge has been cultivated within traditional medical institutions, Buddhist monastic settings, and the households of Thai villages across the country. Some traditional herbals have now been recognized by the Ministry of Public Health as an important facet of the country's national healthcare system. These herbs are used by Thai traditional practitioners in some hospitals in Thailand [3]. In contemporary times, Thailand offers a multitude of traditional remedies that serve for both treating and preventing various symptoms. Notable examples encompass Kleeb Bua Daeng, Triphala, and Ben-Ja-Kaysorn, all widely recognized and frequently employed throughout the nation. The Trikaysornmas formula (TKM) is within this fascinating array of Thai traditional formulations. TKM consists of three medicinal herbs, including *Aegle marmelos* fruit*, Jatropha multifida* bark, and *Nelumbo nucifera* stamen, in the ratio of 1 : 1 : 1. It was employed with intentions related to stimulating appetite, providing a tonic effect, and adaptogenic purpose. In this formula, one gram of TKM formula was added into 200 mL of hot water and taken orally 4 times per day before meal and bedtime [4–6].

From the previous investigation, this formula was reported to have anti-inflammatory and antioxidant activity [4, 5]. Bael or *A. marmelos* is the first herbal plant in the TKM. It belongs to the family Rutaceae. This plant was used in the traditional as antidiarrhea, appetizing, tonic, and enteropathy [7, 8]. For pharmacological activity, Bael has been reported as an antimicrobial, antioxidant, antihyperglycemic, and anticancer [9, 10]. The next herb in TKM involves the stamina of *N. nucifera*, commonly known as the lotus plant. Within this formulation, the stamen is utilized. In the traditional context, it was employed to support the circulatory system, lower blood lipid, and blood glucose levels, as well as diminish oxidative stress compounds within the body [11]. Moreover, many pharmacological activities of this herb are found, such as antioxidant activity, aldose reductase inhibitory activity, and aphrodisiac activity [12–14]. The last plant in the TKM is *J. multifida*, which belongs to the family Euphorbiaceae. Its common name is coral bush. In traditional medication, this plant can be used for tendon pain, tonic, antidiarrhea, and flu-like syndrome [15]. Many pharmacological activities are reported from this plant, such as antileishmanial, antimalarial, and antimicrobial activities [16, 17].

The extraction method poses a significant challenge in the development of novel and innovative herbal formulas [18]. Historically, herbal formulas were commonly administered in the form of powdered formulations, either filled into capsules or used as decoction powders. However, the conventional formulation approach has its drawbacks, as it may not be comfortable for patients to consume. In order to foster the development of innovative formulations, the extraction of herbal plant powders has been explored, allowing for the creation of new and modified forms. Therefore, it is crucial to develop and study extraction techniques that can yield the highest concentration of active compounds for the formulation of herbal remedies. Various extraction techniques have been employed, including liquid-liquid extraction, solid-phase extraction, and ultrasound-assisted extraction, among others [19–21]. To optimize the extraction efficiency and maximize the levels of active compounds, experimental designs such as the Box–Behnken design (BBD) and response surface methodology (RSM) have been utilized. These designs enable the identification and optimization of key variables that influence the extraction process [22–25].

Despite the numerous reports on each individual composition in this formula, there is still insufficient information regarding the pharmacological activities, appropriate extraction techniques, and the active content necessary for confirming its potential in preventing or treating certain symptoms or diseases. The purpose of this study was to investigate the various factors that could influence the extraction process of the TKM formula using RSM and BBD. Additionally, the study aimed to determine the total active content in the TKM extract, analyze important active compounds using high-performance liquid chromatography (HPLC), and investigate the antioxidant activities of TKM.

## 2. Material and Methods

### 2.1. Chemicals and Equipment

Folin–Ciocalteu phenol reagent, quercetin, sodium acetate, *β*-carotene, gallic acid, imperatorin, kaempferol-3-o-glucoside, vitexin, and scopoletin were purchased from Sigma-Aldrich, St. Louis, MO, USA. Organic solvents, hydrochloric acid, and acetic acid were purchased from Merck Ltd, Darmstadt, Germany, and BDH (VWR International Ltd, USA), and RCI Labscan, Thailand. Sodium carbonate (from Loba Chemie, India), potassium chloride (from QRëC, New Zealand), and aluminum chloride (from Ajax Finechem, Australia) were employed in this study. A high-performance liquid chromatography system (Agilent Technologies, USA) and microplate reader (PerkinElmer Inc, USA) were utilized in the experiment.

## 3. Methods

### 3.1. Optimization of Extraction Method Using Experimental Design

The extraction condition was determined utilizing the response surface methodology (RSM) factorial design, specifically the Box–Behnken Design (BBD). BBD was chosen in this study due to using fewer required runs than the other normal factorial technique. This approach aimed to identify the key factors that influence extraction efficiency. Three factors, solvent concentration, extraction times, and material-to-solvent ratio, were differed at three levels as shown in Table 1 and monitored for four responses: percentage extraction yield, total flavonoids, total phenolic, and antioxidant activity using DPPH. The preceding responses underwent optimization via multiple response algorithms using software (Design Expert, version 13). Seventeen experimental designs were created based on the individual parameters, each having three levels, as illustrated in Table 1. The utilization of RSM and BBD was intended to optimize parameters, analyze the impacts of all factors, and assess the resulting responses. The linear polynomial equation obtained from the ANOVA is elaborated in the following equation:

(1) $$\begin{array}{c} Y=a_{0}+a_{1}x_{1}+a_{2}x_{2}+a_{3}x_{3}+a_{12}x_{1}x_{2}+a_{13}x_{1}x_{3}+a_{23}x_{2}x_{3}+a_{11}x_{1}^{2}+a_{22}x_{2}^{2}+a_{33}x_{3}^{2}, \end{array}$$

where *Y* is the observed response; *a*_0_ is a constant; *a*_1_–*a*_33_ are the regression coefficients computed from the observed experiment; and *x*_1_, *x*_2_, and *x*_3_ are the coded values of independent variables denoting the solvent concentration, material-to-solvent ratio, and the extraction times, respectively.

### 3.2. Determination of Total Active Contents in TKM Extract

#### 3.2.1. Preparation of TKM Extract

TKM powder was extracted and macerated for 7 days with 95% ethanol at room temperature. The crude extracts were stored at −20°C prior to analysis.

#### 3.2.2. Quantification of Total Phenolic Content

The determination of phenolic compound in the extract of TKM employed the Folin–Ciocalteu method, following the modified protocol as well as the methodology presented by Ngamkhae in 2021 and Blainski in 2013 [22, 26]. For a concise overview, 20 *µ*l of extract solution (prepared by dissolving 1 mg of extract in 1,000 *µ*l of ethanol) was introduced to a mixture comprising 10% Folin–Ciocalteu reagent (100 *µ*l) and 7% sodium carbonate (80 *µ*l). After a 30-minute incubation period in the absence of light, the absorbance was recorded at 760 nm using a microplate reader. Quantification was achieved by expressing it as milligram gallic acid equivalents per gram of extract (mg GAE/g extract).

#### 3.2.3. Quantification of Total Flavonoid Content

The quantification of flavonoid within the extract was carried out utilizing the aluminum chloride method [22]. In a succinct description, we combine 20 *µ*l of the extract solution with 15 *µ*l of aluminum chloride, 20 *µ*l of 10% sodium acetate, and 145 *µ*l of distilled water. Following a 15-minute incubation period in darkness, the absorbance value was gauged at 430 nm using a microplate reader. The quantification of the total flavonoid was determined and presented as milligrams of quercetin equivalents per gram of the extract (mg QE/g extract).

#### 3.2.4. Quantification of Total Anthocyanin Content

The assessment of total anthocyanin was executed utilizing the pH differential method, following the procedure outlined by Ngamkhae and coworkers in 2021 [22]. Twenty *µ*l of the extract was mixed with either 100 *µ*l of 0.025 M potassium chloride solution (at pH 1) or 100 *µ*l of 0.4 M sodium acetate solution (at pH 4.5). Subsequently, the absorbance was measured at both 535 and 700 nm using a microplate reader. In conveying the findings, the content of total anthocyanin was indicated as milligrams of cyanidin-3-glucoside equivalents per gram of the extract (mg C3G/g extract).

#### 3.2.5. Quantification of Total Alkaloid Content

The quantification of total alkaloid within the extract of TKM was performed using a modified version of the method originally outlined by Shamsa and coworkers in 2008 [27], involving the utilization of bromocresol green (BCG) as an reagent [28]. To determine, one hundred milligrams of each extract was dissolved in 2 mL of 2 N hydrochloric acid, followed by filtration. A one-milliliter sample solution underwent three consecutive extractions with chloroform, and the solution's pH was neutralized using hydrochloric acid. Subsequently, the solution was subjected to a mixture of 5 mL of BCG and 5 mL of phosphate buffer solution at pH 4.7, followed by agitation. Furthermore, the introduction of 10 mL of chloroform facilitated the extraction of alkaloids through vigorous shaking. The resulting extract was collected and diluted with chloroform to achieve a final volume of 10 mL in a volumetric flask. Notably, atropine was employed as the standard reference. The absorbance of the solution was gauged at 470 nm. Expressing the results, the total alkaloid content was denoted as milligram atropine equivalents per gram of the extract (mg AE/g extract).

#### 3.2.6. Determination of Total Carotenoid Content

The quantification of total carotenoid contents followed the method detailed by Ngamkhae and coworkers [22]. Initially, five hundred mg of TKM was dissolved in a mixture of hexane, acetone, and ethanol with a ratio of 2 : 2 : 1. Subsequently, all solutions underwent a 20-minute sonication process, followed by centrifugation at 4°C and 4000 rpm for 10 minutes. The resulting supernatants were combined and mixed with 5 mL of water. This mixture was separated into two distinct layers upon thorough mixing: an upper organic layer and a lower aqueous layer. The organic layer was meticulously collected and then transferred to a 96-well plate (200 *µ*L). To quantify, the absorbance of the solutions was measured at 450 nm employing a microplate reader. In this study, *β*-carotene was employed as the standard across a range of concentrations: 20, 40, 60, 80, 100, and 120 *µ*g/mL. Through the application of the calibration curve equation, the total carotenoid content was computed and expressed as milligrams of *β*-carotene equivalents per gram of extract (mg/*G* extract).

### 3.3. Determination of Active Compounds in TKM by HPLC and Method Validation

#### 3.3.1. Preparation of Standard and Sample Solutions

Five chemical compounds, including imperatorin, vitexin, scopoletin, gallic acid, and kaempferol-3-O-glucoside, were used as standards in the HPLC procedure. Each standard, one milligram in quantity, was dissolved in methanol to prepare a stock solution (1000 *µ*g/mL). The working standard solutions were then prepared by diluting the stock solution with the mobile phase. The analytical standard range was set between 5 and 30 *µ*g/mL.

Ten milligrams of TKM were dissolved in 1 mL of methanol and adjusted to a final volume of 2 mL with the mobile phase. Before injection, the extract solution was filtered through a 0.45 *µ*m filter. The content of active compounds in the extract was determined using standard curves ranging from 5 to 30 *µ*g/mL, which were prepared by diluting the stock solution.

#### 3.3.2. HPLC Condition

The HPLC system was performed using a reversed-phase HPLC equipped with a diode array detector and a Hypersil ODS C18 column (4.0 mm × 250 mm, 5 *µ*m) from Agilent Technologies Inc., Santa Clara, USA. The analytical HPLC conditions were modified from the study conducted by Maneenet and coworkers in 2019 for the determination of active compounds in some herbal products, Kleeb Bua Daeng formula [29]. A volume of 20 *µ*L of the sample was injected for analysis. The detection wavelengths for each standard were set at 254 nm for imperatorin, 260 nm for kaempferol-3-O-glucoside, 275 nm for gallic acid, and 340 nm for scopoletin and vitexin. The mobile phase consisted of acetonitrile (A) and 0.2% acetic acid in water (B), with a flow rate of 1 mL/min. The gradient elution program was as follows: 0–20 min with 85% B, 20–50 min with 82% B, 50–51 min with 50% B, and 51–60 min with 85% B.

#### 3.3.3. Method Validation

The validation of the HPLC method encompasses several key parameters, ensuring its accuracy, precision, limit of detection, limit of quantitation, linearity, range, and robustness. These parameters are systematically assessed and analyzed in accordance with the guidelines laid out by the International Council for Harmonisation (ICH) in ICH Q2 (R2), as well as the guidelines established by the Association of Analytical Communities (AOAC) [30, 31]. This rigorous validation framework serves to substantiate the dependability and trustworthiness of the HPLC method employed in the analytical process.

### 3.4. Evaluating the Antioxidant Activities of TKM

#### 3.4.1. Radical Scavenging Activity Using DPPH Reagent

The method for assessment was carried out in accordance with the procedure documented by multiple researchers [32, 33]. Briefly, the DPPH reagent was prepared by dissolving 7.9 mg of DPPH in 100 ml of ethanol and was stored at −20°C until needed. In the assay, 100 *µ*L of the extract at a concentration of 10 mg/mL and 100 *µ*L of the prepared DPPH reagent were combined in microplate wells. The mixture was allowed to react at room temperature for 30 minutes. After the incubation period, the absorbance was measured at 517 nm utilizing a microplate reader (PerkinElmer Inc: HH3400, USA). A standard curve was established using Trolox as the reference standard, covering concentrations ranging from 10 to 50 *µ*M. The percentage of inhibition of DPPH was calculated using the following formula:

(2) $$\begin{array}{c} \%\text{inhibition}=\left[\frac{\left(A_{\text{DPPH}}-A_{\text{sample}}\right)}{\left(A_{\text{DPPH}}-A_{\text{blank}}\right)}\right]\times 100. \end{array}$$

In this context, *A*_DPPH_ corresponds to the absorbance of the DPPH radical solution (without any sample or standard), while *A*_sample_ denotes the absorbance of the DPPH solution containing the sample or control. Using these values, the inhibitory concentration at 50% (IC_50_) was then determined.

#### 3.4.2. Radical Scavenging Activity Using ABTS Radical Cation Decolorization

This technique was derived from prior published research [32, 33]. The ABTS^•+^ solution was conceived by incubating ABTS with potassium persulfate and allowing it to react in the dark at room temperature for 12 hours. To obtain an absorbance of 0.70 ± 0.02 at a wavelength of 734 nm, 1 mL of the ABTS^•+^ solution was diluted with 50 mL of ethanol. For the experiment, the extract (50 *µ*L) at different concentrations and the ABTS^•+^ reagent (100 *µ*L) were introduced into microplate wells and left at room temperature for a duration of 2 hours. Afterward, the absorbance was assessed at 734 nm. To establish a reference, a calibration curve was constructed using a Trolox standard solution within a range of 10 to 50 *µ*M. The percentage inhibition of ABTS was calculated using the following formula:

(3) $$\begin{array}{c} \%\text{inhibition}=\left[\frac{\left(A_{\text{DPPH}}-A_{\text{sample}}\right)}{\left(A_{\text{DPPH}}-A_{\text{blank}}\right)}\right]\times 100. \end{array}$$

In this context, “*A*” denotes absorbance. Subsequently, the inhibitory concentration at 50% (IC_50_) was calculated.

#### 3.4.3. Oxygen Radical Absorbance Capacity (ORAC)

The oxygen radical absorbance capacity (ORAC) assay is the measurement of the antioxidant capacity of the substance [34]. The ORAC assay measures a fluorescent signal from a probe that is quenched in the presence of reactive oxygen species (ROS). Antioxidants absorb the generated ROS a fluorescent signal to persist Trolox as a standard, which is used to compare with the unknown antioxidants. This method uses AAPH to produce a peroxyl free radical upon thermal decomposition. Free radical making reaction that will be found in the body. The extracts were tested with a fluorescent reagent (150 *µ*l) and mixed with the extract solution 25 *µ*l in 96 black plates. AAPH (25 *µ*l) was added to each well of a 96-well plate, and fluorescence was measured at an excitation wavelength of 485 nm and emission 518 nm.

#### 3.4.4. Ferric Reducing Antioxidant Power (FRAP)

The FRAP reagent was prepared by mixing 300 mM acetate buffer pH 3.6 and TPTZ in 40 mM HCl in the ratio 10 : 1 : 1, respectively. The samples were dissolved in ethanol and mixed with FRAP reagent. The absorbance will be measured at 593 nm. A calibration curve will be plotted by the concentration of Trolox [34, 35].

#### 3.4.5. Metal Chelating Activity

Metal chelating activity was investigated using a modified method described in some reports [36, 37]. Two hundred *μ*L of TKM extracts and their extracts (dissolved in DI water) were combined with 10 *μ*L of 2 mM ferrous chloride solution (FeCl_2_) and 570 *μ*L of distilled water. Twenty *μ*L of 5 mM ferrozine (3-(2-pyridyl)-5,6-diphenyl-1,2,4-triazine-p,p′-disulfonic acid monosodium salt hydrate) was added. Then, the solutions were kept at room temperature for 10 minutes. The absorbance was measured at 562 nm. For correlation, EDTA (ethylene diamine tetra acetic acid) was utilized as a positive control.

In this assay, a sample solution of 50 *µ*L was mixed with 50 *µ*L of 1 mg/mL ferrozine in 1% HCl and 50 *µ*L of FeCl_2_ solution at a concentration of 1 mg/mL. The resulting solution was thoroughly mixed and kept in the dark for 60 minutes. After the incubation period, the absorbance of the solution was measured at 570 nm.

#### 3.4.6. Lipid Peroxidation Assay

Lipid peroxidation was analyzed in a linoleic acid model by ferric thiocyanate method [38, 39]. The TKM extract and its herbal components of 5 mg were dissolved in 50 mM phosphate buffer pH 7.0 (2 mL). Then, linoleic acid (26 *μ*L) and ethanol (2 mL) were added to the solution. To adjust the final volume to 5 mL, distilled water was used. The solution was maintained at 40°C in the dark condition for 1 hour. One hundred *μ*L of 20 mM ferrous chloride (FeCl_2_) in 3.5% hydrochloric acid, 100 *μ*L of 30% ammonium thiocyanate, and 700 *μ*L of 70% ethanol were added into the 200 *μ*L solution. After 3 minutes, the solution's absorbance at 500 nm was measured. *α*-Tocopherol was used as the positive control.

### 3.5. Statistical Analysis

Each experimental group was analyzed in three triplication. Design Expert® (version 13), a statistical software developed by Stat-Ease Inc., based in Minneapolis, MN, USA, was employed to design the experimental conditions for the extraction method.

## 4. Results and Discussion

### 4.1. Experimental Design for Extraction Method

Based on the Box–Behnken design (BBD) experimental setup, seventeen experimental groups were handled involving three factors at three levels, measuring four responses (Table 2). Additional exploration aimed to assess the correlation between the recorded responses and the autonomous factors by using RSM utilizing these data, and a quadratic model was chosen to represent the percentage extraction yield, phenolic and flavonoid content, and the antioxidant activity measured via the DPPH reagent. Throughout this study, three independent factors, the percentage of solvent (ethanol in water, *x*_1_), number of extractions (*x*_2_), and material-to-solvent ratio(*x*_3_), were altered throughout the test runs. The qualitative outcomes were percentage extraction yield (*Y*_1_), total phenolic content (*Y*_2_), total flavonoid content (*Y*_3_), and percentage inhibition of antioxidant activity using DPPH (*Y*_4_) and were systematically varied in the experimental trials. The ANOVA outcomes for *Y*_1_, *Y*_2_, *Y*_3_, and *Y*_4_ demonstrated elevated adjusted *R*^2^ values, underscoring a strong association between the experimental data and the fitted model. Moreover, the high *F* values across all response models indicated the appropriateness of these equation models for predicting the respective responses (Table 3). In the lack-of-fit evaluation, the *P* values for all responses exceeded 0.05, affirming the excellent alignment of the model with the data.

From Table 2, the run order that yielded the highest amount of the important substance or percentage of extraction yield, which was 35.40 ± 0.28%, is run number 2. In this run, a solvent mixture of 95% ethanol and water (40 : 60) was used. The extraction process was carried out three times, and the proportion of herbal substance to the solvent was 1 : 10. In run order 16, using a solvent of 95% ethanol, with 2 times extraction and a herbal material-to-solvent ratio of 1 : 13, the highest amount of the important phenolic compounds obtained was 246.04 ± 6.14 mg GAE/g extract. The run order that yielded the highest amount of the important group of flavonoids, which was 12.75 ± 0.24 mg QE/g extract, is run order 16. In this run, a solvent mixture of 95% ethanol was used with duplicate extraction, and the ratio of herbal material to solvent was 1 : 13. The run order that yielded the highest antioxidant activity with an IC_50_ value of 25.38 *µ*g/mL is run order 9. It utilized 95% ethanol:water (70 : 30) as the solvent and involved a single extraction. The ratio between the herbal material and the solvent was 1 : 13.

In the comprehensive assessment, the significant regression and the lack-of-fit results, which were nonsignificant, indicated that the regression equation effectively illustrated the relationship between the response values (*Y*) and the independent variables (Table 3). The equations (Equations (4)–(7)) were fitted as follows:

Percentage extraction yield:

(4) $$\begin{array}{c} {Y_{1}=26.00-7.20x_{1}-\;3.92x_{2}+1.72x_{3}-1.15x_{1}x_{3}-\;1.75x_{2}^{2}-0.55\;x}_{3}^{2}. \end{array}$$

Total phenolic content:

(5) $$\begin{array}{c} Y_{2}=193.33+54.25x_{1}-0.58x_{2}+7.63x_{3}-15.57x_{1}x_{2}+14.72x_{1}x_{3}+4.08x_{2}x_{3}-40.27x_{1}^{2}-23.61x_{2}^{2}+10.99x_{3}^{2}. \end{array}$$

Total flavonoid content:

(6) $$\begin{array}{c} {\;Y}_{3}=11.73++1.92x_{1}+0.24x_{2}-\;0.32x_{3}-0.04x_{1}x_{2}+0.92x_{1}x_{3}-0.55x_{2}x_{3}-1.48x_{1}^{2}. \end{array}$$

Half-maximal inhibitory concentration of antioxidant activity using DPPH reagent (IC_50_):

(7) $$\begin{array}{c} Y_{4}=31.51-3.53x_{1}-1.36x_{2}-0.41x_{3}-1.82x_{1}x_{3}-0.73x_{2}x_{3}+1.05x_{1}^{2}-2.44x_{2}^{2}-0.79x_{3}^{2}. \end{array}$$

The recorded values of the response data obtained from BBD were utilized in each model to assess them using the coefficient of determination (*R*^2^). This statistical measure represents the proportion of the model that can elucidate the variation observed in the responses.

**Figure 1:**
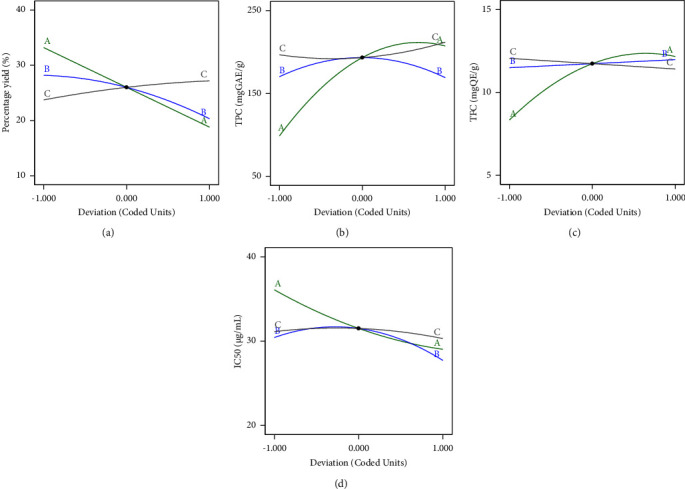
Perturbation plot showing the several factors (line A: percent of ethanol, line B: number of extraction times, and line C: the material-to-solvent ratio) affecting the percentage yield of extracted compounds (a), total phenolic content (b), total flavonoid content (c), and the IC_50_ of antioxidant activity of DPPH (d).

To explore the impact of various variable factors on different responses, a perturbation plot serves as a tool for comparing all factors at specific points within the specified design area. The response of each factor was graphed while altering one factor across its range, holding the other factors constant (refer to Figure 1). Data are deemed responsive to a factor if the line on the perturbation plot exhibits a steep and curved slope. Conversely, if the line appears flat, the response is considered insensitive to changes caused by that specific factor. In our investigation, Line A reflects the most significant change across all responses. Its steep and curved slope indicates that the percentage of ethanol in the extraction solvent notably influenced this response.

From Figure 1(a), the primary factor that has the greatest influence on the amount of extract is the solvent (A), which has a highly curved characteristic with the steepest slope. The secondary factor that has a lesser effect is the ratio between the herbal powder and the solvent (C) and the number of extraction cycles (B), which have a less steep slope compared to the type of solvent.

From Figure 1(b), the primary factor that has the greatest impact on the quantity of the most important phenolic compounds is the type of solvent (Factor A). Specifically, solvents with a steep slope in the graph yield higher quantities of phenolic compounds. The secondary factors that have a lesser impact are the ratio of herbal powder to solvent (Factor C) and the number of extraction cycles (Factor B), as indicated by the lower slopes in the graph compared to the type of solvent.

From Figure 1(c), the primary factor that has the greatest impact on the quantity of important flavonoid compounds extracted is the solvent (A), which has a highly curved shape and the steepest slope. The secondary factors that have a slightly lesser influence are the extraction cycles (B) and the ratio between the herbal powder and the solvent (C). Additionally, it can be observed that the graph has a gentler slope compared to the type of solvent.

From Figure 1(d), the primary factor that has the greatest impact on the antioxidant activity is the type of solvent (A), which exhibits a highly curved shape and the steepest slope the same as Figures 1(a), 1(b), and 1(c). The secondary factors that have a slightly lesser influence are the extraction cycles (B) and the ratio between the herbal powder and the solvent (C). Additionally, it can be observed that the graph has a gentler slope compared to the type of solvent, indicating a lesser impact on the antioxidant activity.

Therefore, the factor that has the greatest impact on the efficiency of TKM extraction in this study, looking at the percentage yield, TPC, TFC, and antioxidant activity of DPPH, is the type of solvent with varying quantities of ethanol. These findings verified that the response model accurately portrayed the anticipated extraction process. The desirability approach for finding an optimal balance between the responses was performed. The overall desirability function (D) was 0.607, which was the average of the individual desirability functions of each response.

### 4.2. Determination of Total Active Contents

Five kinds of total active compounds (phenolic, flavonoids, anthocyanins, alkaloids, and carotenoids) were determined in this study, and the results are shown in Table 4. Based on the study, the highest amount of the important compound was phenolics, followed by flavonoids, alkaloids, carotenoids, and anthocyanins, in that order. Among these active compounds might play a key role for the biological activity of this TKM formula. Therefore, according to the study, substantial amounts of these important compounds could be immensely advantageous and had the potential to be applied in exploring the therapeutic properties of this TKM remedy.

### 4.3. Determination of Five Active Compounds in TKM Formula by HPLC and Validation Results

High-performance liquid chromatography (HPLC) was used in the determination of five active compounds in this study. HPLC plays a role in the analysis of active compounds in many studies [40–42]. Before using, HPLC was developed and validated to ensure its reliability. The HPLC method was developed and validated in this study. The development of a method for analyzing important active compounds using HPLC requires validation to ensure that the method is reliable and can be used accurately to quantify various important substances. The validation results are presented in the table below (Table 5). Based on the results of the validation parameter tests presented in the table, it was found that the HPLC analytical method in this instance has met the acceptance criteria defined by the ICH guidelines. The amount of these compounds is shown in Table 6. These five active compounds including gallic acid, imperatorin, kaempferol-3-O-glucoside, scopoletin, and vitexin can serve as important markers for quality control in TKM formulations and extract. These substances are crucial components found in various types of medicinal preparations within TKM, and they have been extensively studied and researched, showing promising biological properties.

### 4.4. Determination of Antioxidant Activities

Drawing from studies investigating various methods to assess antioxidative effects, the findings highlighted in Table 7 demonstrate that the TKM herbal extract showcases robust antioxidant activity. The exceptional antioxidant activity demonstrated by the TKM herbal extract might be attributed to various underlying mechanisms. The array of essential active compounds present in TKM formulations could potentially elicit their effects through diverse mechanisms or work synergistically to deliver antioxidant benefits. The antioxidant activity operates through diverse processes aimed at counteracting oxidative stress and shielding against damage induced by free radicals. Free radicals are characterized by atoms, molecules, or ions possessing unpaired electrons, rendering them highly reactive within chemical compounds. Some common mechanisms of antioxidant activity include free radical scavenging, in which antioxidants neutralize free radicals by donating electrons to discard the unpaired radical or donating hydrogen atoms [43]. Moreover, activation of antioxidant enzymes, in which antioxidants can activate the enzyme (such as superoxide dismutase) to convert dangerous superoxide radicals into less reactive species, is one of the important mechanisms. Antioxidants may facilitate the breakdown of hydrogen peroxide into water and oxygen that can prevent the formation of hydroxyl radicals [43]. The binding of antioxidants with some metal ions can prevent the oxidative reaction. The inhibition of lipid peroxidation, one of the mechanisms, can protect cell membranes and prevent cellular damage. Some synthetic antioxidants are not preferred to use for pharmacological purposes due to toxicological concerns. The role of natural antioxidants from plants exerts high benefit to use, which may protect cellular damage and lower the high risk of chronic diseases. Some phenolic antioxidants have been well defined as chemo-preventive [44]. Phenolic compounds play a significant role in acting as antioxidants through various pathways, with one of their key mechanisms being free radical scavenging. These compounds effectively capture and neutralize free radicals by either accepting or donating electrons or hydrogen atoms. This scavenging activity aids in preventing oxidative damage and offers protection to cells and tissues, thereby reducing the potential harm induced by oxidative stress.

The findings of this study indicate that the TKM extract has potent antioxidant properties, as demonstrated in all conducted experiments. The IC_50_ value for the DPPH assay was found to be 77.50 ± 1.38 *μ*g/mL. In previous studies [4, 5], the EC_50_ values for the DPPH assay were reported to be 9.37 ± 0.82 *μ*g/mL and 3.86 ± 0.11 *μ*g/mL, respectively. Furthermore, the results from Sihanat and coworkers in 2023 [4] evaluated the antioxidant activity using the ABTS assay, yielding an EC_50_ value of 7.91 ± 0.01 *μ*g/mL, and the FRAP assay showed a value of 49.18 ± 0.05 mg Fe(II) per gram of extract. In this study, when assessing the antioxidant activity through the ABTS assay, the IC_50_ value was found to be 25.92 ± 0.44 *μ*g/mL, and the FRAP assay resulted in a value of 0.14 ± 0.00 mg TE/g extract. Notably, the FRAP assay used Trolox as the standard, while the other study used Fe (II) as their standard (4). However, it is worth mentioning that there were no previous reports on the antioxidant activity evaluated through the ORAC, metal chelating, and lipid peroxidation methods in this TKM extract study. Nevertheless, the possibility of the TKM extract exhibiting high antioxidant activity, as observed in previous studies, testing DPPH, ABTS, and FRAP in other plants, such as the fruits of *A. marmelos* [45], the stamina of *N. nucifera* [46], and the bark of *J. multifida* [47], remains promising.

## 5. Conclusion

This study revealed the factors that influence the efficiency of extraction using experimental design, BBD, and RSM and could be developed to get a high content of some active compounds. The highest TPC and TFC were found in the extraction condition with the highest percentage of ethanol, which showed the most influence extraction condition. Total active contents including TPC, TFC, TCC, TAC, and TCC in TKM were reported. Antioxidant activities in various models were revealed and could show the potential of this herb for these activities. An HPLC method was established and validated to analyze the active content present in TKM, a traditional Thai herbal formula. This HPLC procedure was suitable for analyzing certain active compounds found in Thai herbal medicines and TKM, potentially expanding its application in quality control aspects and might be applied to determine these contents in other formulations.

## Figures and Tables

**Table 1:** BBD for the three-level variable factors affecting the extraction of TKM formula.

Run order	Variable factors (code)			Variable factors		
	Solvent (%ethanol [95%] in water)	Number of extraction time	Ratio between material and solvent	Solvent (%ethanol [95%] in water)	Extraction time	Material-to-solvent ratio
1	1	−1	0	100	3	1 : 10
2	−1	−1	0	40	3	1 : 10
3	1	1	0	100	1	1 : 10
4	0	−1	−1	70	3	1 : 7
5	1	0	−1	100	2	1 : 7
6	0	0	0	70	2	1 : 10
7	−1	0	−1	40	2	1 : 7
8	−1	0	1	40	2	1 : 13
9	0	1	1	70	1	1 : 13
10	−1	1	0	40	1	1 : 10
11	0	0	0	70	2	1 : 10
12	0	0	0	70	2	1 : 10
13	0	0	0	70	2	1 : 10
14	0	−1	1	70	3	1 : 13
15	0	1	−1	70	1	1 : 7
16	1	0	1	100	2	1 : 13
17	0	0	0	70	2	1 : 10

**Table 2:** The results obtained from the experimental values and those obtained from approximating the values using the equation used by varying different variables used in the extraction.

Run order	Percentage extraction yield (% yield)		Total phenolic content (mg gallic acid equivalent/g extract)		Total flavonoid content (mg quercetin equivalent/g extract)		Antioxidant activity using DPPH (IC_50_)	
	Observed	Predicted	Observed	Predicted	Observed	Predicted	Observed	Predicted
1	20.20	20.57	200.65	199.86	11.53	11.97	27.45	27.95
2	35.40	35.47	65.63	60.22	8.00	8.06	34.78	35.01
3	13.30	13.22	162.16	167.57	12.17	12.38	26.06	25.23
4	26.60	26.30	177.75	177.75	10.79	11.27	29.71	29.32
5	17.60	17.53	195.18	195.97	12.23	11.57	30.28	30.47
6	26.10	26.12	187.92	193.33	12.15	11.73	31.33	31.51
7	29.40	29.62	111.49	116.89	9.86	9.58	34.02	33.89
8	35.30	35.37	103.50	102.71	6.70	7.10	36.90	36.71
9	21.60	21.90	191.85	191.85	11.08	11.11	25.38	25.78
10	27.50	27.12	89.40	90.19	8.79	8.62	32.20	32.29
11	26.10	26.12	191.85	193.33	11.13	11.73	32.43	31.51
12	26.40	26.12	197.60	193.33	12.04	11.73	31.63	31.51
13	25.30	26.12	195.72	193.33	12.23	11.73	30.73	31.51
14	29.40	29.25	178.65	184.84	11.92	11.72	30.30	29.96
15	17.80	17.95	174.62	168.43	12.14	12.85	27.72	28.06
16	18.90	18.67	246.06	240.66	12.75	12.77	25.87	26.01
17	26.70	26.12	193.58	193.33	12.13	11.73	31.42	31.51

**Table 3:** The statistical outcomes from the ANOVA of the response surface model for the percentage extraction yield (*Y*_1_), total phenolic content (*Y*_2_), total flavonoid content (*Y*_3_), and antioxidant activity of DPPH (*Y*_4_).

Responses	*P* value Model equation	*F* value Model equation	*P* value Lack of fit	*F* value Lack of fit	Predicted *R*^2^	Adjust *R*^2^
*Y*_1_	<0.0001^∗^	378.75	0.5675^∗∗^	0.90	0.9873	0.9930
*Y*_2_	<0.0001^∗^	110.61	0.0841^∗∗^	4.71	0.9105	0.9840
*Y*_3_	<0.0001^∗^	20.95	0.2883^∗∗^	1.83	0.7247	0.8972
*Y*_4_	<0.0001^∗^	51.59	0.4663^∗∗^	1.09	0.8190	0.9620

^∗^Significant at *P* value less than 0.05, ^∗∗^Not significant at *P* value more than 0.05.

**Table 4:** Total active contents and percentage extraction yield in TKM extract.

Total active content	Amount
Total phenolic content (mg GAE/g extract)	303.19 ± 4.09
Total flavonoid content (mg QE/g extract)	20.28 ± 0.16
Total anthocyanin content (mg C3G/g extract)	1.03 ± 0.09
Total alkaloid content (mg atropine/g extract)	13.06 ± 0.05
Total carotenoid content (mg *β*-carotene/g extract)	1.49 ± 0.03

**Table 5:** Validation results for the analysis of gallic acid, imperatorin, kaempferol-3-O-glucoside, scopoletin, and vitexin by HPLC method.

Parameters	Imperatorin	Kaempferol-3-O-glucoside	Gallic acid	Scopoletin	Vitexin
Range (*μ*g/mL)	5–30	5–30	5–30	5–30	5–30
Linearity					
(i) Coefficient of determination (*R*^2^)	0.9985	0.9970	0.9980	0.9998	0.9992
(ii) Regression equation	*Y* = 58.891*x* − 67.837	*Y* = 51.134*x* + 12.887	*Y* = 47.824*x* + 34.08	*Y* = 82.822*x* − 67.837	*Y* = 54.823*x* − 69.077
Percentage recovery (%)	100.04–109.96	85.07–103.52	88.01–101.78	100.27–101.66	97.81–100.37
Precision (% RSD)					
(i) within-day	0.46–0.97	0.13–0.74	0.24–0.70	0.06–0.99	0.21–0.69
(ii) between-day	0.17–1.53	0.37–2.55	0.40–1.81	0.24–1.54	0.30–0.95
Limit of detection (*μ*g/mL)	0.5	0.5	0.25	0.5	0.5
Limit of quantitation (*μ*g/mL)	1.0	2.0	1.0	1.0	1.0
Robustness testing (%RSD)	0.34–0.45	0.35–0.93	0.23–0.34	0.24–0.45	0.78–1.84
Retention time (minute)	47.86 ± 0.11	21.07 ± 0.25	2.58 ± 0.01	9.14 ± 0.06	10.98 ± 0.31
Wavelength detection (*λ*, nm)	254	260	275	340	340

**Table 6:** The content of gallic acid, imperatorin, kaempferol-3-O-glucoside, scopoletin, and vitexin in TKM formula using HPLC.

Active compounds	Content (mg/g extract ± SD, *n* = 3)
Imperatorin	8.68 ± 0.07
Kaempferol-3-O-glucoside	5.75 ± 0.03
Gallic acid	1.56 ± 0.54
Scopoletin	0.05 ± 0.00
Vitexin	1.43 ± 0.00

**Table 7:** Antioxidant activity of TKM formula.

Antioxidant activities	TKM formula (*n* = 3)
DPPH-IC_50_ (*μ*g/mL)	77.50 ± 1.38^*∗*^
ABTS-IC_50_ (*μ*g/mL)	25.92 ± 0.44^*∗*^
ORAC (mmol of TE/g extract)	1.34 ± 0.03^*∗*^
FRAP (mg TE/g extract)	0.14 ± 0.00^*∗*^
Metal chelating (% inhibition at 10 mg/mL)	61.35 ± 3.10^*∗*^
Lipid peroxidation (% inhibition at 5 mg/mL)	19.43 ± 0.07^*∗*^

^*∗*^Significance at *p* value <0.001.

## Data Availability

The data used to support the study are included in the article.

## References

[1] Tabish S. A. (2008). Complementary and alternative healthcare: is it evidence-based?. *International Journal of Health Sciences*.

[2] Yuan H., Ma Q., Ye L., Piao G. (2016). The traditional medicine and modern medicine from natural products. *Molecules*.

[3] Liu C. X. (2021). Overview on development of ASEAN traditional and herbal medicines. *Chinese Herbal Medicines*.

[4] Sihanat A., Rittaisong N., Chaichamnong N. (2023). *In vitro* antioxidants activities of extract of tri-kasorn-mas remedy and its plant ingredients. *Creative Science*.

[5] Phuaklee P., Dechayont B., Chunthorng-Orn J., Itharat A. (2019). Antioxidant, anti-allergic and anti-inflammatory activities and total phenolic compounds of Tregaysornmas formula. *Thammasat Medical Journal*.

[6] National list of essential medicines. https://kpo.moph.go.th/webkpo/tool/Thaimed2555.pdf.

[7] Monika S., Thirumal M., Kumar P. R. (2023). Phytochemical and biological review of *Aegle marmelos* Linn. *Future Science OA*.

[8] Gupta D., Pp J., Kumar P., Jain J. (2018). Evaluation of antioxidant activity of unripe *Aegle marmelos* corr. Fruits. *Journal of Applied Pharmaceutical Sciences and Research*.

[9] Vinita Bisht N., Johar V. (2017). Bael (*Aegle marmelos*) extraordinary species of India: a review. *International Journal of Current Microbiology and Applied Sciences*.

[10] Swarnkar R., Singh D., Choudhary A., Anand S., Rathore A., Jediya H. K. (2019). Pharmacological properties of *Aegle marmelos*: a review. *International Journal of Current Microbiology and Applied Sciences*.

[11] Chen H.-W., Yang M.-Y., Hung T.-W., Chang Y.-C., Wang C.-J. (2019). *Nelumbo nucifera* leaves extract attenuate the pathological progression of diabetic nephropathy in high-fat diet-fed and streptozotocin-induced diabetic rats. *Journal of Food and Drug Analysis*.

[12] Li C., He Y., Yang Y. (2021). Antioxidant and inflammatory effects of *Nelumbo nucifera* gaertn. Leaves. *Oxidative Medicine and Cellular Longevity*.

[13] Temviriyanukul P., Sritalahareuthai V., Promyos N. (2020). The effect of sacred lotus (*Nelumbo nucifera*) and its mixtures on phenolic profiles, antioxidant activities, and inhibitions of the key enzymes relevant to Alzheimer’s disease. *Molecules*.

[14] Paudel K. R., Panth N. (2015). Phytochemical profile and biological activity of *Nelumbo nucifera*. *Evidence-based Complementary and Alternative Medicine*.

[15] de Carvalho C., Vieira Mariano L., S Negrão V., Passarelli Gonçalves C., Cristina Ribeiro Marcucci M. (2018). Phenols, flavonoids and antioxidant activity of *Jatropha multifida* L. Collected in pindamonhangaba, sao paulo state, Brazil. *Journal of Analytical & Pharmaceutical Research*.

[16] Anani K., Adjrah Y., Améyapoh Y. (2016). Antimicrobial, anti-inflammatory and antioxidant activities of *Jatropha multifida* L. (Euphorbiaceae). *Pharmacognosy Research*.

[17] Committee for Compiling a Reference Handbook of Thai Herbal Medicine (2019). FIN TON. *Journal of Thai Traditional & Alternative Medicine*.

[18] Bommakanti V., Puthenparambil Ajikumar A., Sivi C. M. (2023). An overview of herbal nutraceuticals, their extraction, formulation, therapeutic effects and potential toxicity. *Separations*.

[19] Bitwell C., Indra S. S., Luke C., Kakoma M. K. (2023). A review of modern and conventional extraction techniques and their applications for extracting phytochemicals from plants. *Scientific African*.

[20] Abubakar A. R., Haque M. (2020). Preparation of medicinal plants: basic extraction and fractionation procedures for experimental purposes. *Journal of Pharmaceutical and Bioallied Sciences*.

[21] Murokore B. J., California P. V., Wacoo A. P. (2023). Effect of extraction period on total phenolics, total flavonoids, and antioxidant capacity of Ugandan *Camellia sinensis* (L) kuntze, black primary grades and green tea. *Journal of Food Quality*.

[22] Ngamkhae N., Monthakantirat O., Chulikhit Y. (2021). Optimized extraction method for Kleeb Bua Daeng formula with the aid of the experimental design. *Journal of Chemistry*.

[23] Ferreira N., Viana T., Henriques B. (2023). Application of response surface methodology and box–behnken design for the optimization of mercury removal by Ulva sp. *Journal of Hazardous Materials*.

[24] Leyva-Jiménez F.-J., Fernández-Ochoa Á., Cádiz-Gurrea M. d l L. (2022). Application of response surface methodologies to optimize high-added value products developments: cosmetic formulations as an example. *Antioxidants*.

[25] Silva E. d. O., Borges L. L., Conceição E. C. d., Bara M. T. F. (2017). Box–Behnken experimental design for extraction of artemisinin from Artemisia annua and validation of the assay method. *Revista Brasileira de Farmacognosia*.

[26] Blainski A., Lopes G. C., de Mello J. C. P. (2013). Application and analysis of the Folin ciocalteu method for the determination of the total phenolic content from limonium brasiliense L. *Molecules*.

[27] Shamsa F., Monsef H., Ghamooshi R., Verdian-rizi M. (2008). Spectrophotometric determination of total alkaloids in some Iranian medicinal plants. *The Thai Journal of Pharmaceutical Sciences*.

[28] Ajanal M., Gundkalle M. B., Nayak S. U. (2012). Estimation of total alkaloid in Chitrakadivati by UV-Spectrophotometer. *Ancient Science of Life*.

[29] Maneenet J., Daodee S., Monthakantirat O. (2019). Kleeb Bua Daeng, a Thai traditional herbal formula, ameliorated unpredictable chronic mild stress-induced cognitive impairment in ICR mice. *Molecules*.

[30] (2022). International Council for harmonisation of technical requirements for pharmaceuticals for human use: ICH harmonised guidelines. *Validation of Analytical Procedures*.

[31] Horwitz W. (2002). AOAC guidelines for single laboratory validation of chemical methods for dietary supplements and botanicals.

[32] Apak R., Özyürek M., Güçlü K., Çapanoğlu E. (2016). Antioxidant activity/capacity measurement. 1. Classification, physicochemical principles, mechanisms, and electron transfer (ET)-Based assays. *Journal of Agricultural and Food Chemistry*.

[33] Monthakantirat O., Chulikhit Y., Maneenet J. (2022). Total active compounds and mineral contents in *Wolffia globosa*. *Journal of Chemistry*.

[34] Apak R., Özyürek M., Güçlü K., Çapanoğlu E. (2016). Antioxidant activity/capacity measurement. 2. Hydrogen atom transfer (HAT)-Based, mixed-mode (electron transfer (ET)/HAT), and lipid peroxidation assays. *Journal of Agricultural and Food Chemistry*.

[35] Benzie I. F. F., Strain J. J. (1996). The ferric reducing ability of plasma (FRAP) as a measure of ‘antioxidant power’: the FRAP assay. *Analytical Biochemistry*.

[36] Wang T., Jónsdóttir R., Ólafsdóttir G. (2009). Total phenolic compounds, radical scavenging and metal chelation of extracts from Icelandic seaweeds. *Food Chemistry*.

[37] Wong F.-C., Yong A.-L., Ting E. P.-S., Khoo S.-C., Ong H.-C., Chai T.-T. (2014). Antioxidant, metal chelating, anti-glucosidase activities and phytochemical analysis of selected tropical medicinal plants. *Iranian Journal of Pharmaceutical Research: International Journal of Psychological Research*.

[38] Memarpoor-Yazdi M., Mahaki H., Zare-Zardini H., Zare-Zardini (2013). Antioxidant activity of protein hydrolysates and purified peptides from Zizyphus jujuba fruits. *Journal of Functional Foods*.

[39] Li Y., Si D., Sabier M., Liu J., Si J., Zhang X. (2023). Guideline for screening antioxidant against lipid-peroxidation by spectrophotometer. *eFood*.

[40] Liaudanskas M., Viškelis P., Jakštas V. (2014). Application of an optimized HPLC method for the detection of various phenolic compounds in apples from Lithuanian cultivars. *Journal of Chemistry*.

[41] Shao X., Zhao J., Wang X., Tao Y. (2018). Rapid screening and quantitative determination of active components in qing-hua-yu-Re-formula using UHPLC-Q-TOF/MS and HPLC-UV. *Journal of Analytical Methods in Chemistry*.

[42] Khismatrao A., Bhairy S., Hirlekar R. (2018). Development and validation of RP-HPLC method for simultaneous estimation of curcumin and piperine. *International Journal of Applied Pharmaceutics*.

[43] Lobo V., Patil A., Phatak A., Chandra N. (2010). Free radicals, antioxidants and functional foods: impact on human health. *Pharmacognosy Reviews*.

[44] Rani R., Arora S., Kaur J., Manhas R. K. (2018). Phenolic compounds as antioxidants and chemopreventive drugs from Streptomyces cellulosae strain TES17 isolated from rhizosphere of *Camellia sinensis*. *BMC Complementary and Alternative Medicine*.

[45] Phuaklee P., Dechayont B., Chunthorng-Orn J., Itharat A. (2018). Anti-allergic, anti-inflammatory and antioxidant activities of *Aegle marmelos* Correa. Fruit. *Thammasat Medical Journal*.

[46] On-nom N., Thangsiri S., Inthachat W. (2023). Seasonal effects on phenolic contents and in vitro health-promoting bioactivities of sacred Lotus (*Nelumbo nucifera*). *Plants*.

[47] Hirota B. C. K., Miyazaki C. M. S., Mercali C. A. (2012). C-glycosyl flavones and a comparative study of the antioxidant, hemolytic and toxic potential of *Jatropha multifida* leaves and bark. *International Journal of Phytomedicine*.

